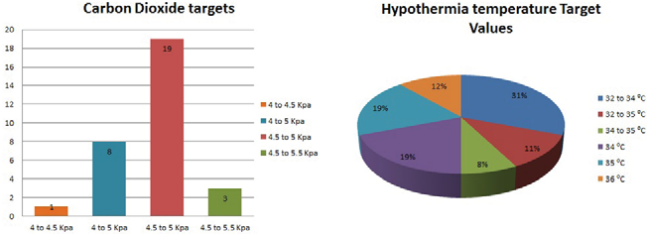# National survey of current protocols and management of the traumatic brain injury patients in UK ICUs

**DOI:** 10.1186/cc12264

**Published:** 2013-03-19

**Authors:** B Lewinsohn, S Panchatsharam, S Wijayatilake, A Billini, G Delacedra, R Jain, J Khan, R Shetty, A Lewinsohn

**Affiliations:** 1 Queens Hospital, Romford, UK

## Introduction

Following primary neurological insult, initial management of traumatic brain-injured (TBI) patients has a clearly defined pathway [1]. However, after arrival at tertiary centers, further management is not standardized. Intracranial hypertension (ICH), systemic hypotension, hypoxia, hyperpyrexia and hypocapnia have all been shown to independently increase mortality [2]. Despite numerous studies, there is currently no level 1 evidence to support any specific management [3]. Our objective was to provide an overview of the current clinical management protocols in the UK.

## Methods

Thirty-one ICUs managing patients with severe TBI were identified from the RAIN (Risk Adjustment In Neurocritical care) study, and a telephone survey was conducted.

## Results

A total 97% of units used a cerebral perfusion pressure protocol for the initial management, with 83% targeting pressures of 60 to 70 mmHg and 17% aimed for >70 mmHg. Ninety-one percent of units monitored CO_2 _routinely with 61% targeting CO_2 _of 4.5 to 5 kPa (Figure 1). Regarding osmotherapy, mannitol was still the preferred agent, with 48% of units using it as first line; 32% used hypertonic saline, while 20% of units used either depending on clinicians' preference. Sixteen percent questioned were currently enrolled on the Eurotherm hypothermia trial, while 16% never used hypothermia and one unit used prophylactic hypothermia routinely. The remaining 65% of units used hypothermia only to manage refractory ICH.

## Conclusion

There is no clear consensus on the initial targets used. The surviving sepsis campaign showed that protocol-led care can reduce mortality [4]. Perhaps it is time for a similar approach to be adopted, with specialists coming to together to review the evidence and formulate guidelines that can then be tested.

**Figure 1:**